# Differential expression of Cosmc, T-synthase and mucins in Tn-positive colorectal cancers

**DOI:** 10.1186/s12885-018-4708-8

**Published:** 2018-08-16

**Authors:** Xiaodong Sun, Tongzhong Ju, Richard D. Cummings

**Affiliations:** 1 Department of Surgery, Beth Israel Deaconess Medical Center, Harvard Medical School, 3 Blackfan Circle, Room 11087, Boston, MA 02115 USA; 2 Department of Biochemistry, Emory University School of Medicine, Atlanta, GA 30322 USA; 3 Office of Biotechnology Products (OBP), Center for Drug Evaluation and Research (CDER), U. S. Food and Drug Administration, Bldg 52/72, Room 2120, 10903 New Hampshire Ave, Silver Spring, MD 20993 USA

**Keywords:** Colorectal carcinoma, T-synthase, Cosmc, Tn antigen, STn antigen, Mutation

## Abstract

**Background:**

The Tn neoantigen (GalNAcα1-*O*-Ser/Thr) is an O-glycan expressed in various types of human cancers. Studies in several Tn-expressing cancer cell lines and pancreatic tumors have identified loss of Cosmc expression caused by either mutations or promoter hypermethylation. In this study, we explored the mechanism(s) for Tn expression in human colorectal cancers (CRC).

**Methods:**

Tn-expressing cell populations were isolated from CRC cell lines by Fluorescence-associated cell sorting (FACS). The expression of the Tn and sialylated Tn (STn) antigens, Cosmc, T-synthase, and mucins was characterized in paired specimens with CRC and in CRC cell lines by immunostaining, western blot, and qPCR.

**Results:**

Using well-defined monoclonal antibodies, we confirmed prevalent Tn/STn expression in CRC samples. However, a majority of these tumors had elevated T-synthase activity and expression of both Cosmc and T-synthase proteins. Meanwhile, Tn antigen expression was not caused by mucin overproduction. In addition, we found that Tn-expressing CRC cell lines had either loss-of-function mutations in *Cosmc* or reversible Tn antigen expression, which was not caused by the deficiency of T-synthase activity.

**Conclusions:**

Our results demonstrate multiple mechanisms for Tn expression in CRCs.

**Electronic supplementary material:**

The online version of this article (10.1186/s12885-018-4708-8) contains supplementary material, which is available to authorized users.

## Background

The Tn neoantigen (GalNAcα1-*O*-Ser/Thr) and its sialylated form (sialyl Tn, STn) are tumor-associated carbohydrate antigens (TACAs) expressed in a broad range of human cancers, including those in the colorectum, breast, prostate, lung, ovary, cervix, and pancreas [1, 2]. The Tn/STn neoantigens have promise as tissue or serum biomarkers in cancer detection and prognosis, and in providing a tumor-specific epitope for targeted therapy [2]. In addition, they are involved in promoting cancer progression or protecting malignant cells from the surveillance of the immune system, hence being valuable therapeutic targets in clinical treatment [3].

Although Tn has been recognized as a neoantigen, few analyses have used paired normal and tumor samples to define its expression [2]. Some studies compared the healthy individuals and the patient group, which may not reflect the progression of the Tn antigen. In addition, the Tn positivity rate varies within a particular cancer type. For example, 68 of 146 (47%) colorectal cancers (CRCs) were reported to be Tn positive, while another study concluded 72–81% [4, 5]. The differences were probably influenced by the detection approaches used, since many studies have used GalNAc-binding lectins, such as *Vicia villosa* agglutinin (VVA) and *Helix pomatia* agglutinin (HPA), or the antibodies that were privately in-house generated and often not extensively characterized for specificity [6]. We have utilized an IgM-type monoclonal antibody BaGs6 (CA3638) to the Tn antigen [7]. BaGs6 specifically recognizes glycoconjugates containing GalNAcα1-*O*-Ser/Thr but not blood group A and related glycans terminating in GalNAc, and BaGs6 also stains tissue sections from mice engineered to express the Tn antigen but does not stain normal tissues [8, 9]. Therefore, BaGs6 is a reliable and well-characterized reagent to explore the Tn positivity in human cancers.

The mechanisms of generating the Tn neoantigen in human cancers are unclear. The Tn antigen is a precursor structure biosynthesized in the Golgi apparatus by a family of twenty different polypeptide α-N-acetylgalactosaminyltransferases (ppGalNAc-Ts), which transfer GalNAc from the donor UDP-GalNAc to a Ser or Thr residue in glycoproteins [10]. In normal tissues, the Tn antigen is usually undetectable due to its efficient conversion into more extended glycans, primarily to the core 1 structure (Galβ1-3GalNAcα1-O-Ser/Thr, the T or TF antigen) [6]. This modification is catalyzed by a single enzyme, T-synthase (C1GALT1, UDP-Gal:GalNAcα1-O-Ser/Thr glycopeptide β3-galactosyltransferase) in the Golgi apparatus [11]. The core 1 structure is further elongated to extended core 1 O-glycans, or branched to core 2 structures, or sialylated [6]. In the gastrointestinal tract (GI tract), GalNAcα1-O-Ser/Thr may be converted into core 3 O-glycans [12]. In addition, the Tn antigen can be sialylated to form STn [6].

The T-synthase is a unique enzyme whose correct folding requires an X-linked molecular chaperone, Cosmc (Core 1 β3-Gal-T-Specific Molecular Chaperone, also named C1GalT1C1) [13]. In the ER, Cosmc interacts co-translationally with non-native T-synthase to generate active T-synthase, which is then transported to and functions in the Golgi apparatus [14]. Defective Cosmc function results in aggregation and proteosomal degradation of T-synthase associated with expression of the Tn antigen [13]. Studies on Tn-expressing cancer cell lines and patients with Tn syndrome revealed loss-of-function mutations in the *Cosmc* gene and loss of T-synthase (Additional file 1). Promoter hypermethylation of *Cosmc* was also identified in Tn-positive human pancreatic cancers and the Tn4 cells, suggesting that reduction of Cosmc and T-synthase contributes to Tn neoantigen expression in human cancers [15, 16].

Here we defined the expression of the Tn and STn antigens and characterized Cosmc and T-synthase in matched CRC specimens and in several CRC cell lines. We conclude that expression of the Tn antigen arises from multiple pathways, including mutation of *Cosmc*, as observed in some CRC cell lines such as LS 180 and HCT8, and alternative mechanisms in CRC specimens and the SW480 line.

## Methods

### Human specimens and cancer cell lines

Paraffin-embedded tissue sections from 39 colorectal cancer patients were obtained from the Emory Tissue bank and Dr. N. Volkan Adsay (Departments of Anatomic Pathology, Emory University School of Medicine, Atlanta), and frozen tissues were requested for 27 cases randomly. For each case, both tumor and its matched normal tissue (normal) were analyzed. Transitional mucosa (TM) was visible in the tumor sections of 11 cases, which is immediately adjacent to the cancer and exhibits microscopic abnormalities without atypia [4]. Usage of these specimens was reviewed and approved by the Emory University Institutional Review Board (IRB) with informed consent from patients, and the research team did not receive any identifying patient information.

Human colorectal carcinoma cell lines were purchased from American Type Culture Collection (ATCC) and were cultured following the ATCC instructions:

LS 180- ATCC CL-187; HCT8- ATCC CCL-244; SW480- ATCC CCL-228; SW620- ATCC CCL-227; SW1116- ATCC CCL-233; HCT15- ATCC CCL-225; T84- ATCC CCL-248; Caco-2- ATCC HTB-37 (Research Only); HT29- ATCC HTB-38 (Research Only); None of these cell lines require ethics statements.

Additional ATCC cell line included: HEK293T human embryonic kidney- ATCC CRL-3216; this cell line does not require ethics statements.

LS174T-Tn(−) and LS174T-Tn(+)-II cells were isolated from LS174T cells (ATCC CL-188) [17].

LOX and LSC cells were obtained and used as previously described [17].

Tn4 cells were obtained and used as previously described [16].

### Immunofluorescence

Human CRC cells were cultured in Lab-Tek™ II-chamber slides (Thermo Fisher Scientific, Waltham, MA) for 48 h before fixation in 4% formaldehyde for 15 min at room temperature. After washing in PBS, cells were blocked for 1 h in PBS containing 10% (*v*/v) normal goat serum. Cells were then incubated with the anti-Tn antibody BaGs6 at 4 **°**C overnight followed by Alexa Fluor® 488- or 568-conjugated goat anti-mouse IgM antibody (Invitrogen, Carlsbad, CA) for 60 min at 4 **°**C. After four washes in PBS, nuclei were counterstained with 4′,6-diamidino-2-phenylindole (DAPI) for 5 min. The chamber was then removed, and slides were mounted and imaged with a Zeiss Axioplan 2 fluorescent microscope (Zeiss, Oberkochen, Germany).

### Flow cytometry and fluorescent activated cell sorting (FACS)

Cultured CRC cells were trypsinized, washed, and suspended in cold PBS. One million (1 × 10^6^) cells were incubated with BaGs6 or isotype control (mouse IgM) for 40 min on ice, followed by incubation with Alexa Fluor® 488-conjugated goat anti-mouse IgM secondary antibody. After three washes with cold PBS, cells were analyzed in a Becton Dickinson FACscan flow cytometer (BD Biosciences, San Jose, CA). In FACS, ten to twenty million (1~ 2 × 10^7^) cells were immunostained and sorted into 15 ml tubes by a SORP FACSAria II or into 96-well plates by a MoFlosorter (BD Biosciences).

### Immunohistochemistry (IHC)

Tissue sections were deparaffinized, rehydrated, and washed with water. Antigen retrieval was done by heating slides in a pressure cooker for 3 min in citrated buffer (pH 6.0, 10 mM trisodium citrate). After cooling down at room temperature, tissue sections were incubated with 3% hydrogen peroxide and then blocked with 5% normal goat serum in Tris-buffered saline with 0.1% Tween-20 (TBST). Then tissue sections were incubated with primary antibodies at 4 °C overnight, followed by HRP-conjugated secondary antibodies (KPL Inc., Gaithersburg, MA) at room temperature for 1 h. Primary antibodies used in this study included those against Tn (BaGs6, mouse IgM), STn (TAG-72, mouse IgG), blood group A antigen, mucin 2 (H-300) (Santa Cruz Biotechnology, Dallas, TX), and mucin 1 (Thermo Fisher Scientific). Signals were visualized by incubating sections with Aminoethylcarbazole (AEC) substrate solution (Invitrogen), and cell nuclei were counterstained with hematoxylin (Invitrogen). Whole tissue sections were mounted in CLEAR-MOUNT solution (Electron Microscopy Sciences, Hatfield, PA) and reviewed by microscopy. The signal intensity was indicated by a numerical scale of 0 to 3 (0 = no staining, 1 = weak staining, 2 = moderate staining, and 3 = strong staining), and the percentage of positive cells was estimated. The IHC score (IS) was calculated by multiplying the staining intensity by the percentage of positive cells. A sample is considered to be positive when the immunohistochemistry score is 50 or greater. Statistical analyses were performed using Paired *t* test. The correlations between two antibodies’ IHC were determined using Pearson correlation coefficient (Pearson’s *r*). Representative slides were scanned with VS120 Whole Slide Scanner (Harvard Medical School Neurobiology Imaging Facility), and pictures were captured using the OlyVIA 2.9 software (Olympus, Tokyo, Japan).

### Western blot (WB)

Frozen human CRC tissues and cultured cells were sonicated or lysed in a Hepes buffer containing 0.5% Triton X-100 and protease inhibitors (Roche Diagnostics Corporation, Indianapolis, IN). The protein concentration was determined using a BCA kit (Thermo Scientific, Waltham, MA) following the manufacturer’s instructions. Equal amounts of total protein were separated in SDS-PAGE and transferred onto nitrocellulose membranes. Western blot antibodies included those against Cosmc, T-synthase, β-actin, α-tubulin (Santa Cruz Biotechnology), and the Tn antigen (BaGs6). For human CRCs, each WB band was quantified for its intensity and area with ImageJ, and signal was calculated by multiplying band intensity by the area.

### T-synthase activity assay

T-synthase activity assay was performed following the protocol described previously [18]. Briefly, total cell lysate was incubated with 4-Mu-α-GalNAc (acceptor), UDP-Gal (donor), MnCl_2_, Triton X-100, and O-glycosidase in MES buffer (pH 6.8) at 37 °C for 2 h. Reactions were terminated with a stop solution (1 M Glycine–NaOH, pH 9.6). Relative fluorescence units (RFUs) were measured in a Victor Multiple-Label Counter using umbelliferone mode, e.g., Ex 355 nm and Em 460 nm. The specific activity of T-synthase was calculated by normalizing the total activity by the protein concentration and the incubation time.

### Genomic DNA preparation

Genomic DNA of CRC tissues or cultured cells was prepared from the remaining tissue pellet of the protein extraction. Briefly, the pellet was re-suspended and digested in 1.0 mg/ml of proteinase K at 56 °C overnight. Then genomic DNA was extracted and purified using the DNeasy blood and tissue kit (Qiagen, Valencia, CA). DNA concentrations were determined with a Nanodrop spectrophotometer (Thermo Scientific).

### Loss of heterozygosity (LOH) and mutation analyses

Twenty micrograms of genomic DNA from CRC cell lines and specimens were amplified by PCR reactions. LOH status was determined by analyzing sequencing trace files for allele imbalance of single nucleotide polymorphisms (SNPs) in the *Cosmc* and *T-synthase* genes. Primer sequences and the size of PCR products are listed in Additional file 2. For mutation analyses, the coding regions of *Cosmc* and *B3GNT6* (UDP-GlcNAc:βGal β-1,3-N-Acetylglucosaminyltransferase 6, Core 3 synthase) were amplified, and the primers were 5’-TTCTCCATAGAGGAGTTGTTGC-3′ and 5’-TGTGGTTATACCAGTGCCACC-3′ (*Cosmc*) and 5’-GTTCTGGGAGAGAAGTGACGG-3′ and 5’-TCAGCATGGACATGGTTGGAG-3′ (*B3GNT6*). Mutations were determined by comparing sequences to the reference sequence of *Cosmc* (NM_001011551.2) or *B3GNT6* (NM_138706).

### Total RNA extraction and real-time PCR reactions

Frozen human CRC tissues were mashed in liquid nitrogen, and total RNA was isolated using the RNeasy mini kit (Qiagen) following the manufacturer’s instructions. RNA concentrations were determined with a Nanodrop spectrophotometer (Thermo Scientific). One μg of total RNA was reverse transcribed into cDNA using the SuperScript III first strand synthesis system (Invitrogen). Quantitative PCR reactions were performed with the SYBR Premix Ex Taq™ Kit (Clontech Laboratories, Mountain View, CA) in the StepOnePlus™ Real-time PCR System (Applied Biosystems, Carlsbad, California). Relative fold changes were calculated using the 2^-ΔΔCt^ method, with human *β-Actin* mRNA as the internal control. For each gene, PCR primers were located in different exons to avoid possible interference of genomic DNA contamination. Primer sequences were: 5’-AAGCCGTTCTAGACGCGGGAAA-3′ and 5’-GCTCATGGTGGTGCATTCTA-3′ for *Cosmc*, 5’-GTCACCAGTCCCAAGTCGTC-3′ and 5’-TTCAGCCAGGATTTAGAGGC-3′ for *T*-*synthase*, 5’-GATGGCTCCTGTCTATTTCTTCT-3′ and 5’-ACCCTCTGGCGTCTCCTCCT-3′ for *B3GNT6*, 5’-GGTCCTTGCTTCTGGCTGTC-3′ and 5’-CCTGGGACTTAGGCTTTGC-3′ for *ST6GALNAC1*, 5’-TCTTCTGGCTGCTGCTCC-3′ and 5’-TTCAAATGATGTGGTGTCCCT-3′ for *ST6GALNAC2*, and 5’-CAAGAGATGGCCACGGCTGCT-3′ and 5’-AGGACTCCATGCCCAGGAAGG-3′ for *β-Actin*.

## Results

### Some human CRC cell lines contain Tn-positive cells

To explore molecular mechanisms of Tn neoantigen expression in human CRCs, we examined 8 commonly used CRC cell lines (LS 180, SW480, SW620, SW1116, HCT8, HCT15, T84, and Caco-2) for expression of the Tn and T-synthase. LS174T-Tn(−) and LS174T-Tn(+)-II were used as negative and positive controls for Tn, respectively, and HEK293T was included as an additional negative control [17]. By western blot, the 8 CRC cell lines and HEK293T expressed no Tn antigen, but detectable T-synthase protein (Additional file 3 a). The enzyme activities of T-synthase in these cells were comparable to that in LS174T-Tn(−) (Additional file 3 b). However, by immunofluorescence, we found that three cell lines (LS 180, SW480 and HCT8) had approximately 1~ 2% of cells that were Tn(+) on the cell surface, while SW1116 expressed the Tn antigen surrounding a whole cell colony (Additional file 3 c). Therefore, several human CRC cell lines contain a small percentage of Tn(+) cells, indicating a mixed population of cells.

### Tn-positive (Tn(+)) subpopulations of LS 180 and HCT8 cells harbor a mutant *Cosmc* gene

**Fig. 1:**
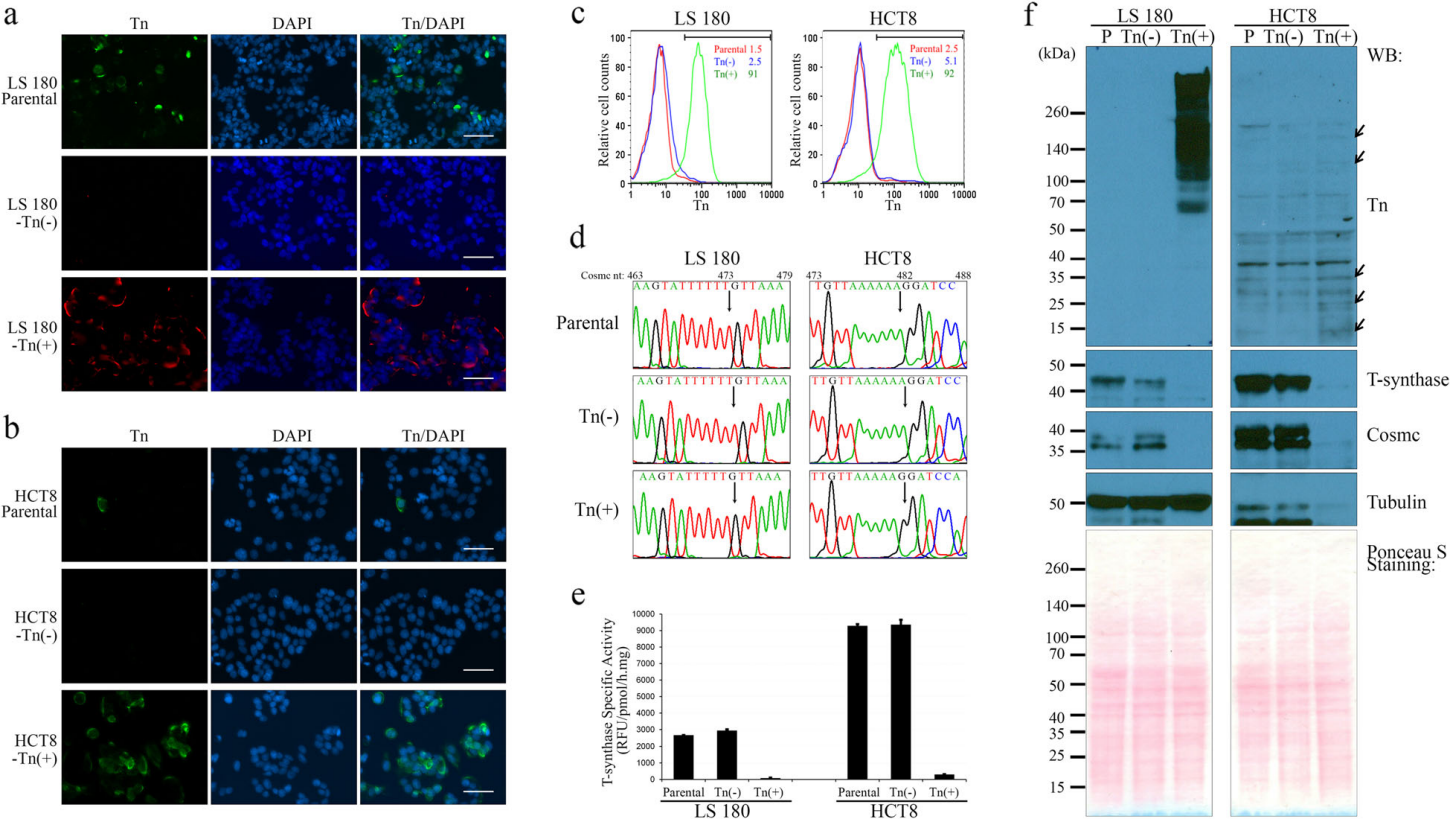
Loss of function of Cosmc in LS 180-Tn(+) and HCT8-Tn(+) cells. **a**, immunofluorescence of the Tn antigen in LS 180 parental, Tn(−) and Tn(+) cells. LS 180 parental cells contained a small number of Tn-positive cells (green). Compared to LS 180-Tn(−) cells, Tn(+) cells expressed robust cell surface Tn antigen (red). **b**, immunofluorescence of the Tn antigen in HCT8 parental, Tn(−) and Tn(+) cells. Compared to HCT8 parental and Tn(−) cells, Tn(+) cells expressed cell surface Tn antigen (green). In **a** and **b**, nuclei were counterstained with DAPI (blue), all scale bars are 50 μm. **c**, flow cytometry analyses of LS 180 and HCT8 subpopulations. Histograms of parental, Tn(−) and Tn(+) cells are shown as red, blue and green lines, respectively. Inset numbers show %Tn(+), defined by the horizontal grey gate. **d**, sequencing analyses of *Cosmc* in LS 180 and HCT8 cells. Parental and Tn(−) cells contained WT *Cosmc* coding region, while LS 180-Tn(+) and HCT8-Tn(+) cells had single nucleotide deletion at nt473 and nt482, respectively. Cell line names are on top, nucleotide positions are labeled above the trace files. The delT473 and delA482 are indicated by arrows. **e**, enzyme activities of T-synthase in LS 180 and HCT8 subpopulations. Compared to the parental and Tn(−) cells, Tn(+) cells had significantly lower enzyme activity of T-synthase. Activity values were determined in triplicates, error bars represent the standard error of the mean (SEM). **f**, expression of the Tn antigen, T-synthase, Cosmc, and α-tubulin in LS 180 and HCT8 cells. LS 180-Tn(+) cells expressed significant amounts of the Tn antigen, whereas HCT8-Tn(+) cells exhibited slightly increased Tn antigen (arrows). There was no detectable T-synthase and Cosmc proteins in both LS 180-Tn(+) and HCT8-Tn(+) cells. Although α-tubulin levels varied in HCT8 subpopulations, Ponceau S staining indicated equal amount of total proteins loaded for WB. Names of cell populations are listed on top. Protein standards are labeled at left, and antibodies at right

By fluorescence-activated cell sorting (FACS), we isolated Tn(−) and Tn(+) subpopulations from LS 180 and HCT8 parental cells using the anti-Tn antibody BaGs6, where only the cells with the strongest fluorescent signal (top 1%) were considered as Tn(+) for collection. For both cell lines, the majority of parental cells (> 97%) were Tn(−). Immunofluorescence (Fig. 1a and b) and flow cytometry (Fig. 1c) analyses confirmed Tn antigen expression in the Tn(+) subpopulations. Furthermore, LS 180-Tn(+) cells expressed the STn antigen at the cell surface, while HCT8-Tn(+) cells did not (Additional file 4).

Genomic sequencing revealed that LS 180- and HCT8-Tn(−) cells contain a wild type (WT) *Cosmc* gene, whereas LS 180-Tn(+) cells harbor a T deletion at nucleotide 473 (delT473), and HCT8-Tn(+) cells have delA482 (Fig. 1d). Both delT473 and delA482 resulted in open reading frame (ORF) shifts and truncated Cosmc proteins (Additional file 1). Accordingly, LS 180- and HCT8-Tn(+) cells, as predicted, exhibited significantly lower T-synthase activities when compared to the parental and Tn(−) cells (Fig. 1e). Western blot demonstrated that LS 180- and HCT8-Tn(+) cells were deficient in Cosmc and T-synthase proteins and acquired Tn expression on several glycoproteins (Fig. 1f). Although the α-tubulin levels varied in different cell populations, staining with Ponceau S indicated equivalent amount of total proteins loaded for WB (Fig. 1f). These results revealed that Tn expression in cancer cell lines is associated with loss-of-function mutations of *Cosmc*.

### Reversible Tn expression in SW480 cells are not due to loss of T-synthase.

**Fig. 2:**
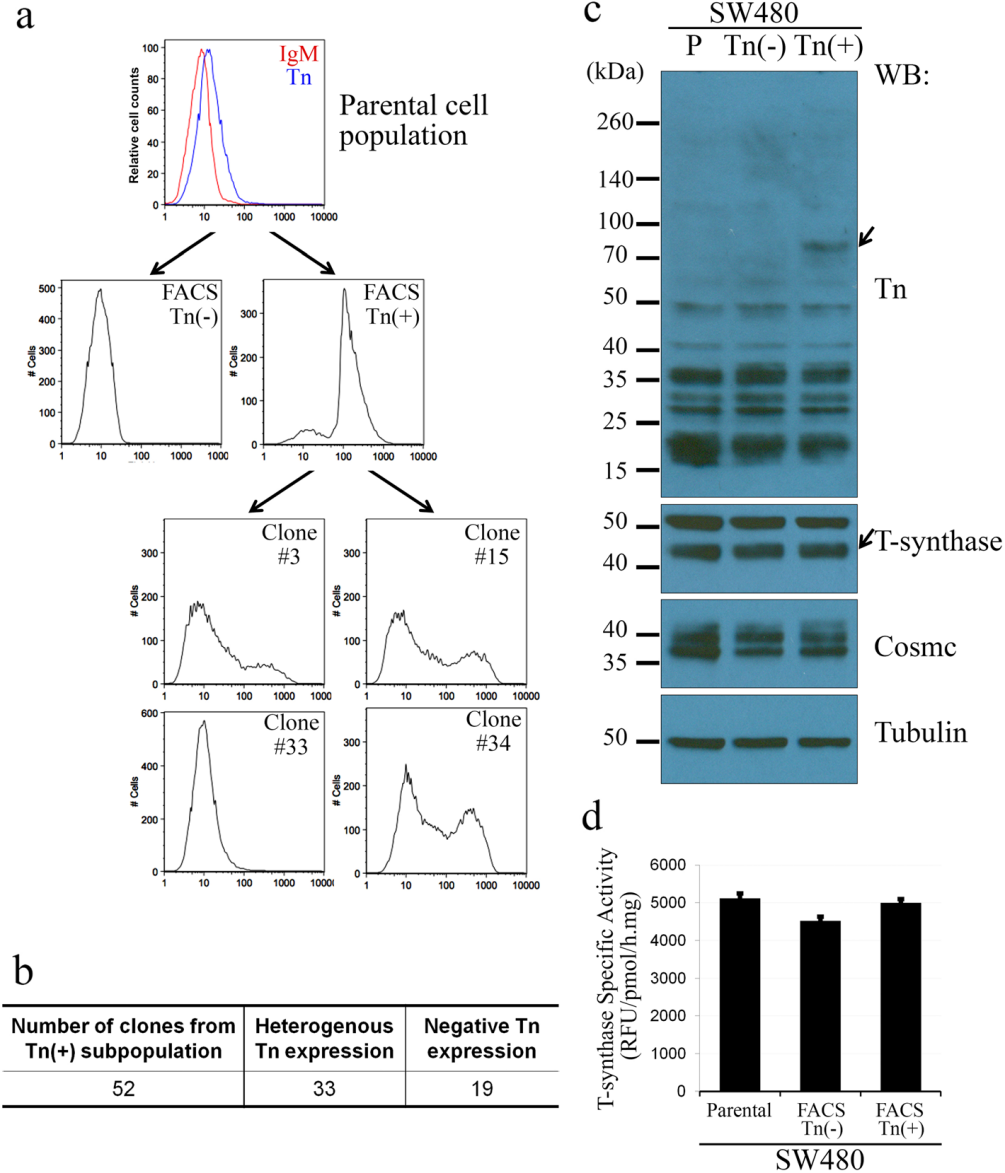
Revertible expression of the Tn antigen in SW480 cells. **a**, flow cytometry analyses of SW480 Tn-positive subpopulation. SW480 parental cells were stained with anti-Tn antibody (BaGs6) and separated into Tn(−) and Tn(+) subpopulations. The Tn(+) subpopulation was sorted into 96-well plates to form single clones. After expansion, these clones were analyzed for Tn expression. While single Tn(+) cells were isolated, their derived clones showed either negative (Clone#33) or heterogeneous (Clone#3, 15 and 34) Tn expression. In parental cells, histograms of isotype control and the BaGs6 staining are shown as red and green lines, respectively. **b**, Summary of Tn-positivity in single cell derived clones. Among a total of 52 clones examined, 19 are negative for the Tn antigen, 33 have a portion of Tn(+) cells, and none expresses the Tn antigen homogenously. **c**, expression of the Tn antigen, T-synthase, Cosmc, and α-tubulin in SW480 cells. SW480-Tn(−) and -Tn(+) cells were separated by FACS and extracted for total proteins. SW480-Tn(+) cells showed additional band representing the Tn-bearing protein(s) (arrows). There were comparable levels of T-synthase and Cosmc proteins in SW480-Tn(+), Tn(−) and parental cells. Names of cell populations are listed on top. Protein standards are labeled at left, and antibodies at right. **d**, T-synthase activities in SW480 subpopulations. Both Tn(−) and Tn(+) cells have comparable T-synthase activities with the parental cells. Activity values were determined in triplicates, error bars represent the standard error of the mean (SEM)

We conducted several FACS experiments on SW480 cells. As shown in Fig. 2a for SW480, the Tn(+) cells were remarkably enriched (85%) after sorting and collecting the top 1% of positive cells. However, we were unable to maintain the Tn(+) subpopulations. The majority of cells were Tn(−) after 3–4 weeks of expansion. We then sorted single SW480-Tn(+) cells into 96-well plates and obtained single-cell-derived clones (Fig. 2a), A total of 52 individual clones were analyzed for cell surface Tn expression, Among them, 19 (37%) clones were Tn(−), and 33 (63%) clones became heterogeneous for Tn expression (Fig. 2b). Representative histograms of Tn expression of these clones are shown in Fig. 2a. Tn heterogeneity developed from the single-cell derived clones demonstrates that Tn expression in SW480 cells is reversible.

We then investigated whether transient expression of the Tn antigen in SW480 cells was caused by temporary reduction of T-synthase or Cosmc. However, western blot showed that SW480-Tn(+) cells had comparable levels of T-synthase and Cosmc to the parental and Tn(−) cells (Fig. 2c). Enzymatic activity assay further indicated that these Tn(+) cells remained a similar level of T-synthase activity as to the Tn(−) cells (Fig. 2d). Hence, unlike LS 180- and HCT8-Tn(+) cells, the transient Tn expression in some SW480 cells were not due to absence or reduction of T-synthase activity. While changes in methylation status can create changes in expression levels, the comparable protein levels coupled with previous data [16] showing that mutation or hypermethylation of *Cosmc* impairs T-synthase activity, did not indicate a role for methylation in the reversibility that we observed.

### Prevalent Tn and STn neoantigen expression in human colorectal cancers

**Fig. 3:**
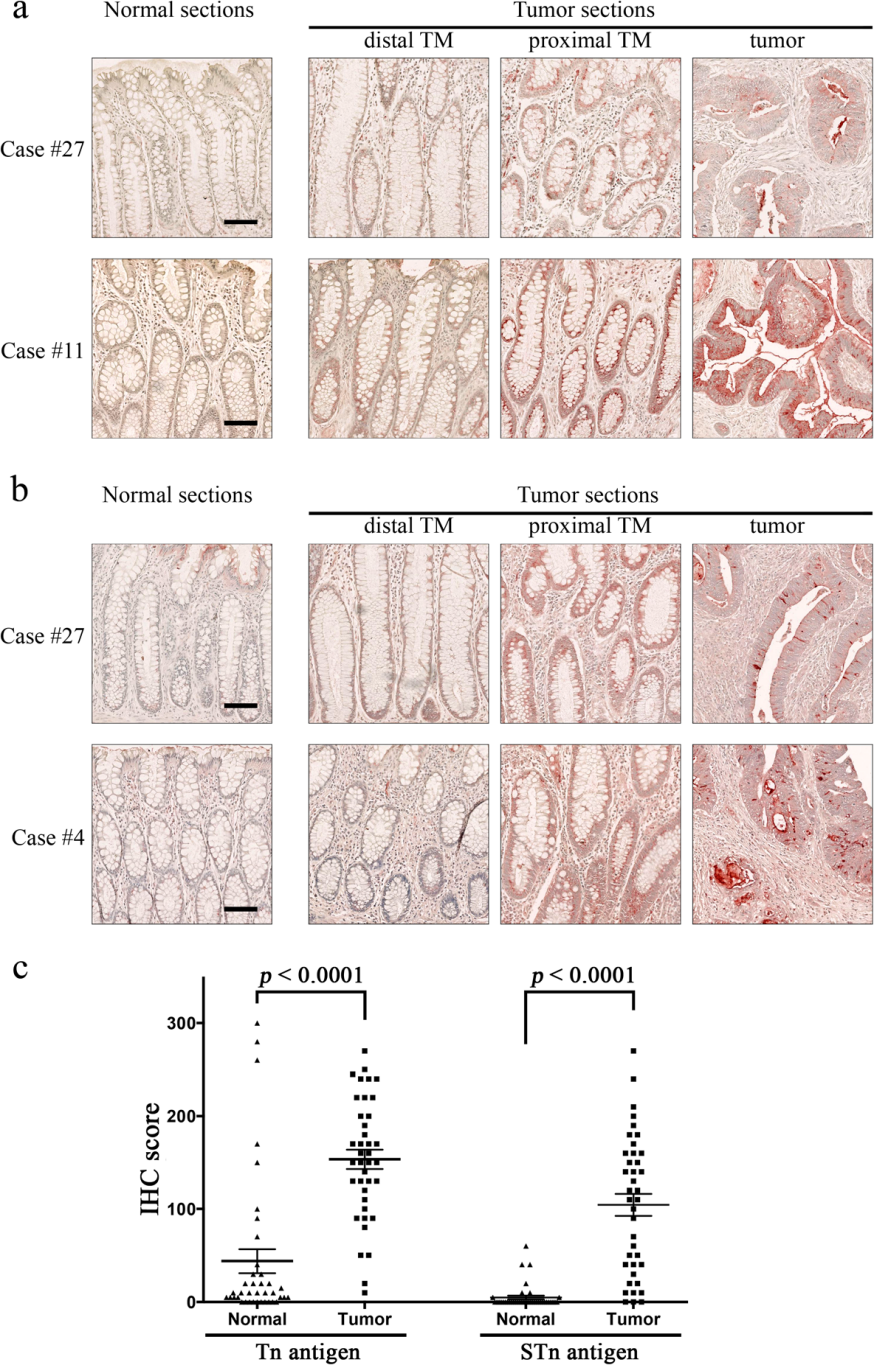
Expression of the Tn and Sialyl-Tn (STn) neoantigens in human CRCs. **a**, representative immunohistochemistry (IHC) of the Tn antigen in 2 cases of matched normal and tumor specimens. **b**, representative IHC of STn in 2 cases of CRCs. In both **a** and **b**, “distal TM” and “proximal TM” denote transitional mucosa (TM) located far and near to the malignant cells, respectively. Compared to the matched normal sections and “distal TM” crypts, the “proximal TM” crypts had increased intracellular Tn and STn antigens. All scale bars are 100 μm. **c**, IHC score (IS) of Tn and STn levels in normal and tumor sections. IS of each staining was plotted with triangle (normal) and square (tumor) shaped boxes respectively. The mean and standard error (SEM) values are indicated by longer lines and shorter lines, respectively. The *p*-values were generated using Paired sample *t* test

Using BaGs6, we examined both tumor and matched adjacent normal tissues (assigned as normal) from the same individual of a cohort of 39 CRC cases to determine Tn neoantigen status. Thirty-seven out of 39 (95%) of the tumors examined had detectable Tn antigen on the epithelial cell surface (Fig. 3a). Meanwhile, 7 (18%) of the adjacent normal tissues were Tn(+); the remaining normal sections had either weak intracellular or absent Tn antigen (Additional file 5). The blood group A antigen (BGA), which contains a terminal α-GalNAc, is often a confounding antigen in studying Tn expression, since some reagents may cross-react with BGA. Therefore, we stained the CRC samples with an anti-BGA antibody and observed distinct staining patterns from those observed for BaGs6 (Additional file 6). All cases are either negative for BGA staining or for those that were positive, staining was observed in blood vessels and red blood cells, while BaGs6 stained epithelial cells. BaGs6 does not recognize the BGA antigen, as shown in prior studies on its specificity, which is consistent with the data here. The tumor sections from 11 patients contained transitional mucosa (TM) regions, which are uninvolved histological “normal” crypts adjacent to the atypical cells. Notably, 7 out of the 11 proximal TMs had gradually increased intracellular Tn antigen, compared to the distal TM in the same section and the matched normal sections (Fig. 3a).

These CRC tissues were also analyzed for the STn antigen using a monoclonal antibody TAG72. We observed robust cell surface STn antigen in most tumors, but rarely was it expressed in matched normal sections (Fig. 3b). All STn-expressing tumors were also Tn positive (Additional file 5). Similar to the Tn neoantigen, increased intracellular STn was observed in the transitional mucosa (TM) (Fig. 3b).

Statistical analyses of the IHC score (IS) showed significant higher levels of cell surface Tn and STn antigens in tumors (both *p* <  0.0001) (Table 1, Fig. 3c). The IS for Tn expression in normal tissue was 43.85 (±12.76) compared to tumor tissue with an IS of 153.46 (±10.40). STn expression in normal tissue was 4.87 (±2.10) compared to tumor tissue IS of 104.36 (±11.86).

**Table 1:** Tn, Sialyl-Tn (STn) antigens, mucin 1 (MUC1), and mucin 2 (MUC2) expression in human colorectal cancers

	n	IS in matched normal (SEM)	IS in tumor (SEM)	*P* value^a^
the Tn antigen	39	43.85 (12.76)	153.46 (10.40)	< 0.0001
the STn antigen	39	4.87 (2.10)	104.36 (11.86)	< 0.0001
MUC1	20	78.50 (10.57)	135.25 (17.77)	0.0074
MUC2	6	300.00 (0.00)	83.33 (36.30)	0.0019

^a^*P* values were determined by the paired *t*-test

*n*, numbers of case examined for IHC; IS, IHC score; *SEM*, standard error of the mean

### Lack of somatic mutations of *Cosmc* and *B3GNT6* in human CRCs

We then analyzed the genomic sequence (single exon) of *Cosmc* in 27 CRC samples (Case #1–27). Unlike what we observed for most Tn-expressing cell lines, no somatic mutation was detected in the single coding exon of *Cosmc*. One tumor harbored a 1-bp deletion in a poly(G) consecutive sequence located in the promoter region, and its effect on *Cosmc* expression remains unknown. In the GI tract, in addition to the core 1 glycan, the epithelial cells also modify the Tn antigen to form the core 3 structure [1]. Therefore, we also analyzed the coding exon of the core 3 enzyme, *B3GNT6* (UDP-GlcNAc:βGal β-1,3-N-Acetylglucosaminyltransferase 6) in the 27 cases of CRCs. No mutation was identified in *B3GNT6*, suggesting the two key glycosyltransferases in the core 1 and core 3 pathways are rarely mutated in CRCs. In addition, no putative CpG islands were identified in the *B3GNT6* promoter, exon1, or intron1 by publically available predictive tools, therefore we did not test the methylation status of *B3GNT6*.

### Loss of heterozygosity (LOH) of the *Cosmc* locus in human CRCs

**Fig. 4:**
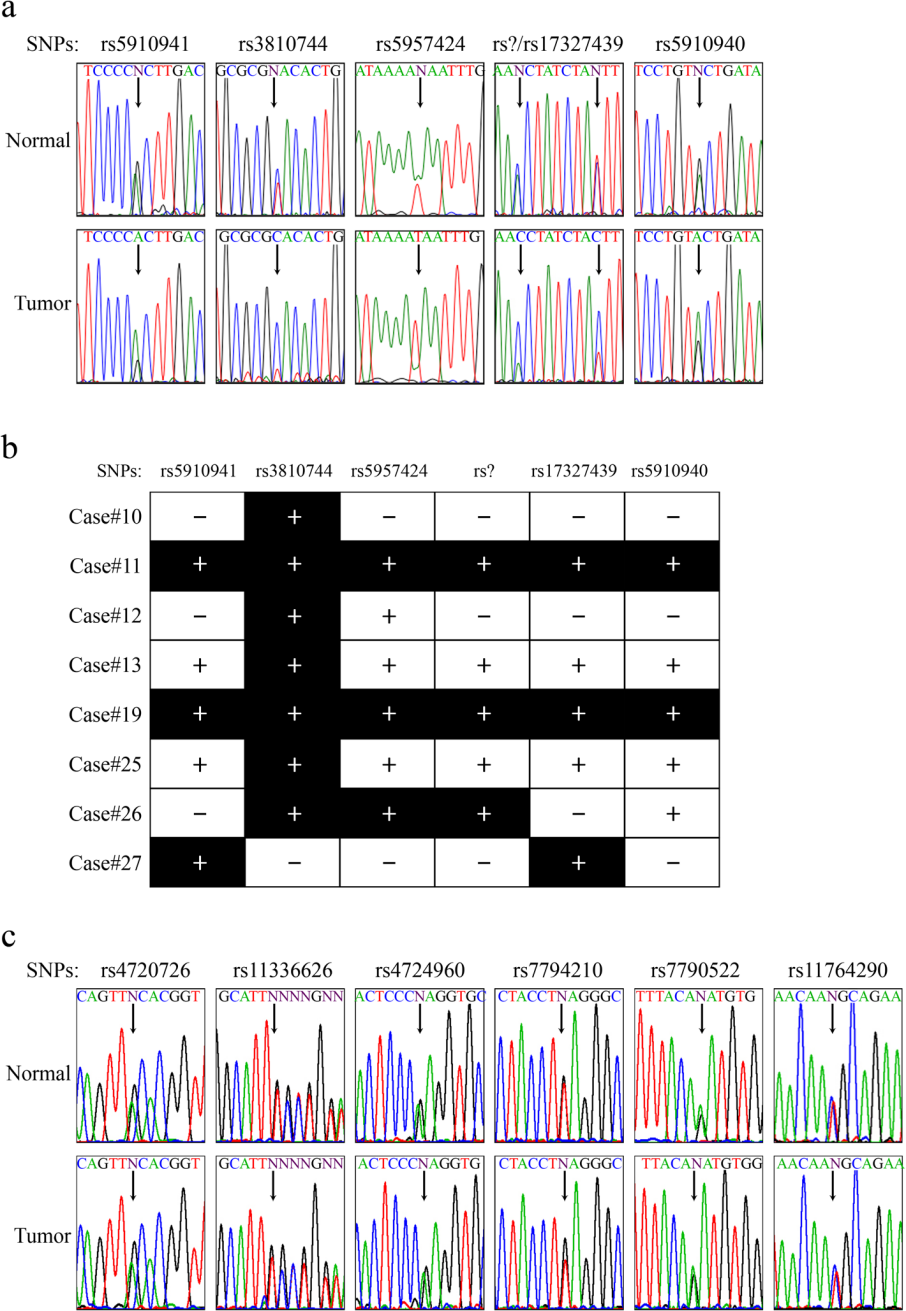
Loss of heterozygosity (LOH) of *Cosmc* and *T-synthase* in human CRCs. **a**, representative allele imbalance of *Cosmc* determined by single nucleotide polymorphism (SNP) combined PCR sequencing. SNP IDs are listed at the top in the order of their relative localizations in the *Cosmc* gene, and SNPs are indicated by arrows. Almost equal peak heights of SNPs were observed in the matched normal tissue, while one allele dramatically decreased in the tumor. “rs?” represents a previously undefined SNP. **b**, summary of the CRC samples with LOH in *Cosmc*. SNP IDs are listed on top, and the case IDs at left. Allele imbalance is indicated by black boxes. The plus and minus mark heterozygosity and homozygosity of a SNP in the normal tissue, respectively. **c**, LOH analyses of *T*-*synthase*. Representative SNP-PCR sequencing was shown in both adjacent normal and tumor specimens. SNP IDs are listed on top in the order of their relative localizations in the *T*-*synthase* gene, and SNPs are indicated by arrows. Almost equal peak heights of SNPs were observed in normal and tumor tissues

LOH is a common mechanism for gene loss of function in tumorigenesis. We investigated the LOH status of *Cosmc* and *T*-*synthase* using the SNP-based PCR approach. Six and 13 SNPs of *Cosmc* and *T-synthase,* respectively, are located at different genomic regions and were manually selected (Additional file 2). Since *Cosmc* is located on the X chromosome (Xq24), LOH could be assessed only in females. In normal tissues two alleles generated equal amounts of PCR products, while in tumors the allele with LOH produced less product at that location (Fig. 4a). Among 15 female CRCs examined, 8 (53%) showed LOH of *Cosmc* within at least one SNP (Fig. 4b and Table 2). None of the cancer specimens examined contained LOH within the *T*-*synthase* (Fig. 4c). Thus, LOH occurred in *Cosmc*, but not the *T-synthase*, in a majority of specimens examined.

**Table 2:** Summary of LOH, expression, and enzymatic activity of Cosmc and T-synthase in human colorectal cancer samples

Gene	LOH	Change	mRNA level	Protein level	Enzymatic activity
Cosmc	8/15 (53%) positive	Increase	8/15 (53%)	18/24 (75%)	N/A
		Decrease	2/15 (13%)	3/24 (12%)	
		No change	5/15 (33%)	3/24 (12%)	
T-synthase	0/24 (0%) positive	Increase	12/15 (80%)	15/24 (63%)	14/24 (58%)
		Decrease	2/15 (13%)	2/24 (8%)	1/24 (4%)
		No change	1/15 (7%)	7/24 (29%)	9/24 (38%)

*LOH*, loss of heterozygosity. *N/A*, not applicable

### Increased *T-synthase* and *Cosmc* expression in human CRCs

**Fig. 5:**
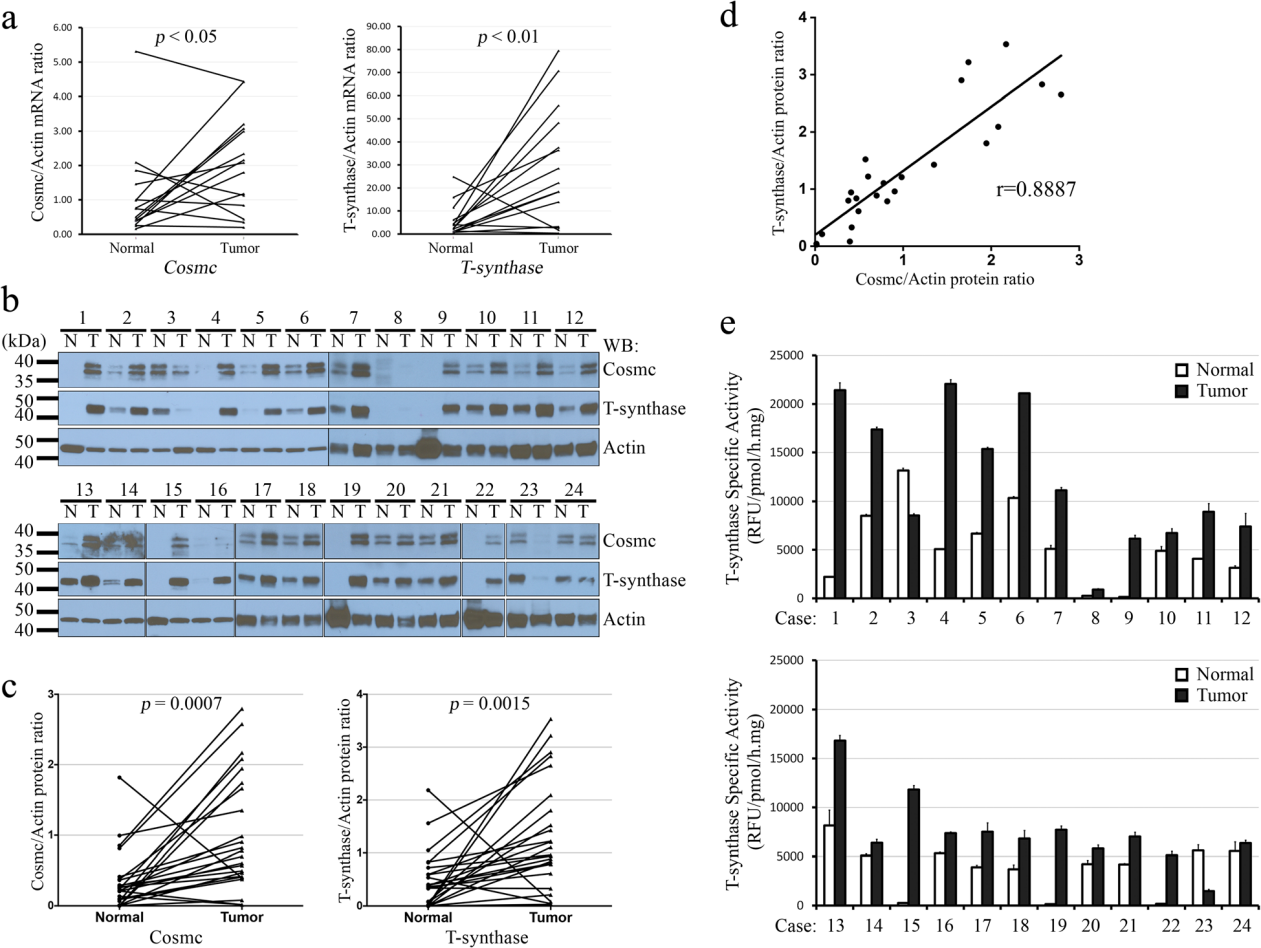
Expression of Cosmc and T-synthase in human CRCs. **a**, mRNA levels of *Cosmc* and *T*-*synthase*. Relative mRNA levels were calculated using the 2^-ΔΔCt^ method, with human *β-Actin* mRNA as internal control. Paired samples are connected by a solid line. *Cosmc* and *T*-*synthase* mRNA levels were significantly higher in the tumors (*p <* 0.05 and < 0.01, respectively). **b**, expression of Cosmc and T-synthase proteins in human CRCs. Case numbers are listed on top. Molecular weight is indicated at left, and protein names at right. Cosmc and T-synthase protein levels were elevated in the majority of tumors (T) compared to the matched normal tissues (N). **c**, relative expression levels of Cosmc and T-synthase proteins in CRCs. Cosmc and T-synthase protein levels detected by WB (**b**) were quantified using ImageJ and normalized to the *β-*Actin protein. Paired samples are connected by a solid line. Cosmc and T-synthase protein levels were elevated in the tumors (*p* = 0.0007 and = 0.0015, respectively). **d**, correlation of Cosmc and T-synthase protein levels in human CRCs. Twenty-four tumors were plotted for their Cosmc/Actin and T-synthase/Actin ratios. Correlation was determined using Pearson Correlation Coefficient, and *r* = 0.8887 (*p* < 0.0001). **e**, T-synthase enzyme activities in matched human CRC samples. Fourteen of 24 cases had increased T-synthase activities in the tumor (> 2 fold change) compared to the matched normal control. White and black bars represent normal and tumor tissues, respectively. Case numbers are listed at the bottom of each panel. Error bars represent the standard error of the mean (SEM) calculated from triplicate

To explore whether there are changes in Cosmc or T-synthase expression in human CRC, we examined *Cosmc* and *T-synthase* in CRC samples at both mRNA and protein levels. Total RNA from paired frozen tissues of 15 patients was subjected to real-time RT-PCR analyses. With a fold-change cut off of 2-fold, *Cosmc* mRNA levels increased in 8 of 15 tumors, compared to that in the matched normal tissues (*p* <  0.05), and *T*-*synthase* mRNA levels increased in 12 of 15 (*p* <  0.01, Fig. 5a and Table 2). The transcript levels of *B3GNT6* in most of these samples were undetectable, making it difficult to draw a clear conclusion.

Moreover, of 24 cases (Case #1–24) examined for protein expression by WB, the majority had increased Cosmc and T-synthase protein levels in the tumor compared to the normal, and only two (Case #3 and #23) showed a decrease in both protein levels in the tumor; Case #8 had decreased Cosmc but not T-synthase levels (Fig. 5b). After quantification of the blots with ImageJ, 18 of 24 cases exhibited elevated Cosmc/β-Actin ratio in the tumor at > 2-fold change, and the Cosmc protein level in overall tumors are higher than that in the normal (*p* = 0.0007, Fig. 5c). T-synthase protein levels showed similar alterations (*p* = 0.0015, Fig. 5c). Interestingly, the Cosmc protein level correlated with that of T-synthase, as determined by Pearson correlation coefficient analysis (*r* = 0.8887) (Fig. 5d).

Furthermore, correlated to the protein levels detected by WB, a majority of the tumor samples had increased enzyme activities of T-synthase, compared to the matched normal tissues (Fig. 5e and Table 2). The two tumors (Case #3 and #23) that had reduced Cosmc and T-synthase proteins also exhibited decreased T-synthase activities. Therefore, in Tn-expressing CRCs, it cannot be simply defined by a loss of T-synthase or loss of enzyme activity, although it should be noted, that our analyses cannot assess the proper localization of the enzyme in the Golgi apparatus, where it normally functions, as the anti-T-synthase and anti-Cosmc antibodies are not suitable for immunohistochemistry.

### Tn antigen expression was not correlated with expression of MUC1 or MUC2

**Fig. 6:**
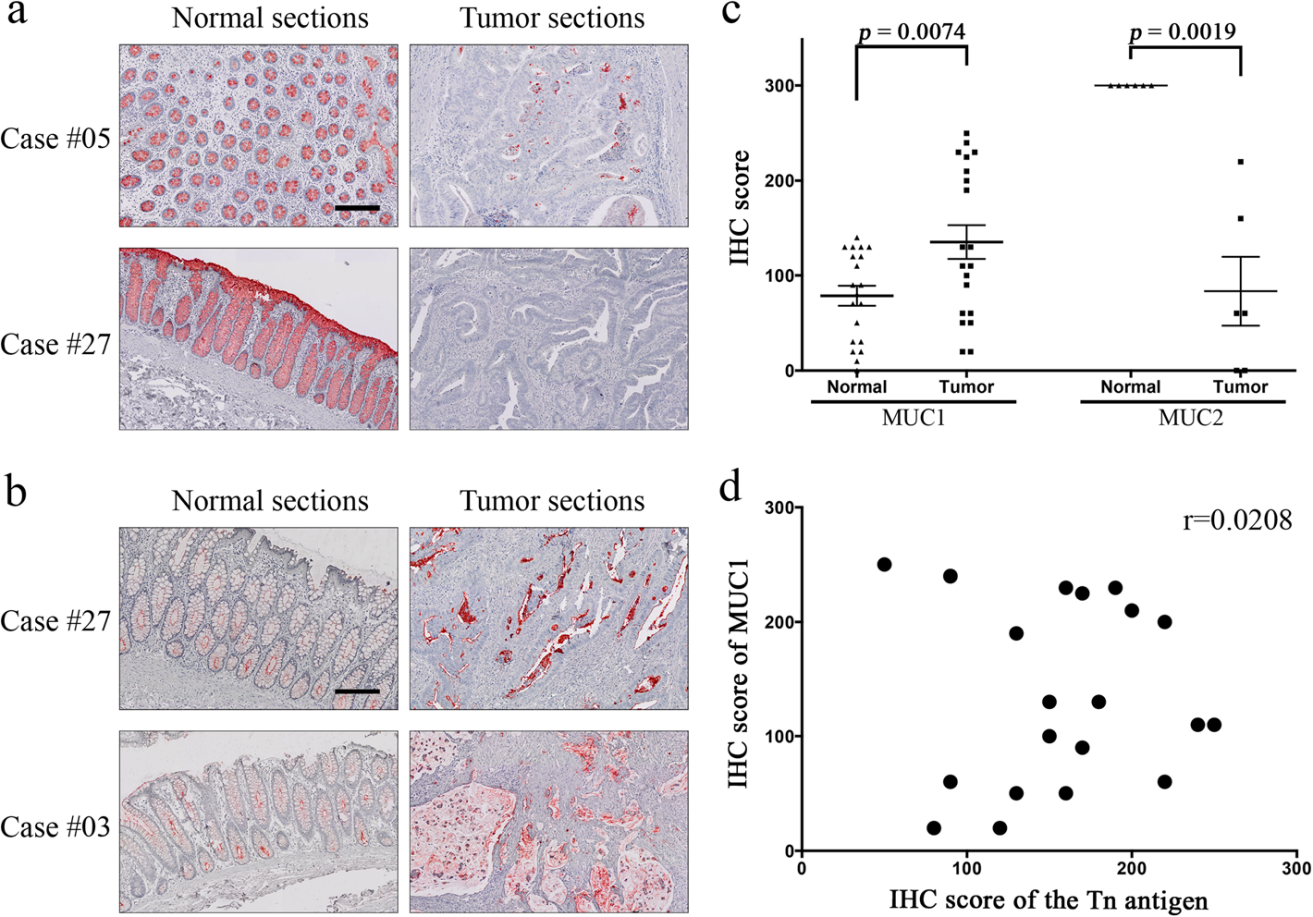
Expression of mucin 2 (MUC2) and mucin 1 (MUC1) in human CRCs. **a**, representative IHC of MUC2 in CRCs. Abundant MUC2 proteins were produced in normal crypts, while reduced or absent expression was observed in tumor sections. **b**, representative IHC of MUC1 in CRCs. Elevated MUC1 proteins were detected in tumor sections. All scale bars are 200 μm. **c**, differential expression of MUC1 and MUC2 in matched CRCs. IHC score (IS) of each staining was plotted with triangle (normal) and square (tumor) shaped boxes respectively. The mean and standard error (SEM) values are indicated by longer lines and shorter lines, respectively. The *p*-values were determined by Paired sample *t* test. **d**, correlation between MUC1 and Tn expression in human colorectal tumors. The IS of 20 tumors were plotted for MUC1 and Tn staining. The Pearson’s *r* was 0.0208, indicating no correlation between MUC1 and Tn antigen expression

Mucins are heavily O-glycosylated and often differentially expressed in tumors [19]. We considered the possibility that overproduction of mucins may result in insufficient modification of terminal α-GalNAc on mucins by T-synthase, thus leading to Tn expression. To test this possibility, we used IHC to define expression of two major mucins produced in the GI tract, MUC1 and MUC2 (Fig. 6a and b). Polyclonal antibodies were used to exclude the variables that may affect recognition of the epitopes. While the gel-forming MUC2 was abundant in normal epithelium, it was remarkably reduced or absent in the tumor (*p* = 0.0019, Fig. 6c, Table 1). MUC1 was significantly elevated in tumor sections (*p* = 0.0074, Fig. 6c, Table 1), but its expression level did not correlate with the IS of the Tn antigen (*r* = 0.0208, Fig. 6d). Hence, we observed no positive association between the MUC1 or MUC2 levels and level of Tn antigen expression.

### Increased *ST6GALNAC1* mRNA levels in human CRCs

**Fig. 7:**
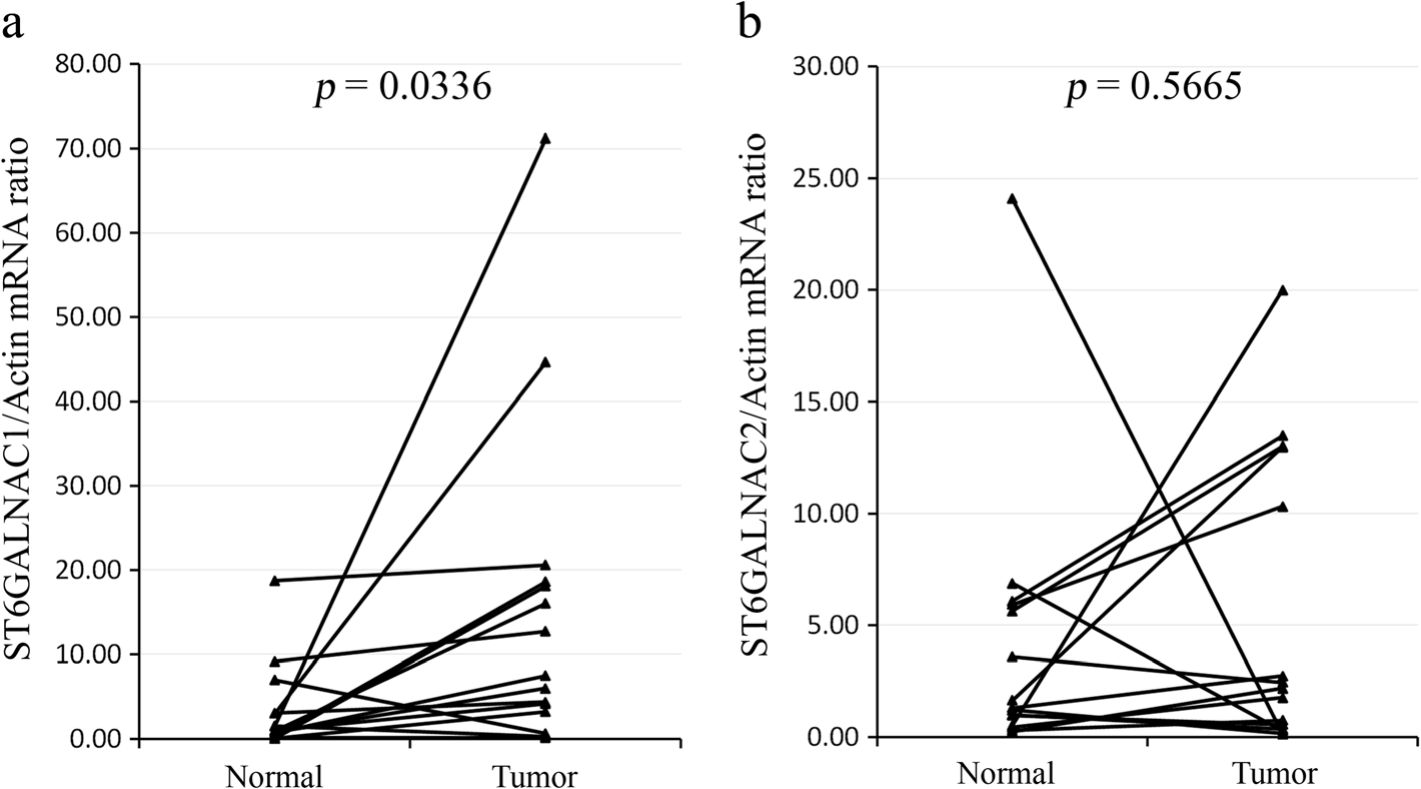
Transcriptional expression of *ST6GALNAC1* and *ST6GALNAC2* in human CRCs. **a**, *ST6GALNA1C* mRNA levels. **b**, *ST6GALNAC2* mRNA levels. Relative mRNA levels were calculated using the 2^-ΔΔCt^ method, with human *β-Actin* mRNA as the internal control. Paired samples are connected by a solid line. The *ST6GALNAC1* mRNA level was significantly higher in the tumors. The significance of the difference between normal and tumor samples (*p* value) was calculated by paired *t* test

Like the Tn neoantigen, the STn antigen was observed to frequently elevate in the CRC specimens, and all STn-bearing tumors were Tn-positive (Additional file 5). ST6GALNAC1 is the enzyme that is required for generating the STn antigen [20]. To determine whether the STn antigen in CRCs was due to increased expression of sialyltransferases [20], we measured the transcripts of *ST6GALNAC1* and *ST6GALNAC2* by quantitative RT-PCR. Nine of 15 tumor samples tested had remarkably elevated *ST6GALNAC1* mRNA levels, but not *ST6GALNAC2* (*p* = 0.0336 and 0.5665 respectively, Fig. 7). Our results support that elevation of the ST6GALNAC1 level is responsible for STn expression in human CRC.

## Discussion

In this study, using specific monoclonal antibodies and multiple approaches, we analyzed matched CRC specimens and several cancer cell lines for expression of the Tn/STn antigens and the core 1 biosynthetic pathway. Our study is unique as it focuses on the progressive expression of Tn in individual colorectal cancer patients [2] and, to our knowledge, it is the first to investigate expression and enzyme activities of the Cosmc/T-synthase pathway in human CRC specimens. Our results showed an increase of intracellular and cell surface Tn antigen in TM regions and tumors, respectively. It not only supports previous reports that both the Tn and STn antigens are prevalent in CRC samples, but also suggests that appearance of intracellular Tn and STn could be an early event during colorectal tumorigenesis. A few other studies also demonstrated the existence of the Tn antigen in gastrointestinal nonmalignant lesions, such as polyps and aberrant crypt foci (ACFs) [21, 22]. Therefore, the Tn and STn antigens could be potential biomarkers for prediction or early detection of human CRCs.

Although the Tn antigen is prevalent in a variety of human cancers, only a few cancer cell lines robustly express the Tn antigen, including Jurkat, Tn4, LSC and LOX [13, 16, 17]. LS174T and HT-29 have a small population of Tn(+) cells [17, 23]. In this study we identified four additional CRC cell lines having a subpopulation of Tn-expressing cells (Additional file 3). Although LS 180 and SW1116 were reported to be Tn positive, the mechanisms for the Tn expression are unclear [24, 25]. Here we identified frame shift mutations of *Cosmc* in LS 180-Tn(+) and HCT8-Tn(+) cells. The mechanistic explanation for this may be that several *Cosmc* mutations in cancer cell lines occur in repeated DNA sequences (microsatellites), suggesting that mononucleotide repeat tracts in the *Cosmc* coding region may be susceptible to the microsatellite instability (MSI) phenotype, like Bax and TGFβRII [26]. The cells with MSI are deficient in DNA mismatch repair and therefore exhibit higher mutation rates in microsatellites [26]. Indeed, LS174T, LS 180 and HCT8 are MSI cells [27]. This may explain the observation that *Cosmc* mutations in LS 180-Tn(+) and HCT8-Tn(+) cells were identical to that in Jurkat and LS174T-Tn(+)-II respectively, although they were derived from different patients; LSC, LS174T and LS 180 originated from the same patient but harbored different mutations. In addition to LS 180 and HCT8, we identified that Tn expression in SW480 was unstable and did not result from loss or reduction of T-synthase activity. These observations suggest that, while loss of functional Cosmc by mutation, deletion, or hypermethylation contributes to Tn expression in some cancer cell lines and certain types of malignancies (such as pancreatic cancer) observed so far [13, 17, 23, 28, 29], other mechanisms do exist to cause revertible expression of the Tn antigen. This may underlie the paradox that while the Tn antigen is a common marker of many tumors in situ, few tumor-derived cell lines express the Tn antigen.

Unlike most of Tn-expressing cell lines, we did not detect any *Cosmc* somatic mutations in 27 cases of CRCs. Similarly, lack of *Cosmc* mutations were also reported in colorectal cancer in other studies [30, 31]. Moreover, although a number of CRC specimens examined harbored LOH at the *Cosmc* locus, few expressed reduced levels of *Cosmc* and *T-synthase*. Conversely, the majority of the tumors had increased Cosmc and T-synthase mRNA and protein levels (Fig. 5). Since *Cosmc* is located on the X chromosome, our results suggest that these LOH-occurring samples had likely kept the active allele of *Cosmc*, which was further transcribed at a higher level in cancer cells. Elevated enzyme activities of T-synthase in these samples indicate that the enzyme is correctly folded, which is known to require functional Cosmc. Interestingly, the Cosmc protein levels in the CRC samples were well correlated with T-synthase (Fig. 5d), suggesting co-expression of the two proteins may be regulated in a coordinate fashion, consistent with studies on the promoter elements in the *Cosmc* and *T-synthase* genes [32]. Up-regulation of the *T-synthase* mRNA in CRCs was observed in several gene profiling studies, although the Tn antigen status in these samples was not determined [33].

The molecular mechanism underlying elevated Cosmc and T-synthase expression in Tn-positive tumors is unclear, but there are several possibilities to consider in future work. As a molecular chaperone in the ER, Cosmc may be induced by ER stress in cancer cells. ER stress commonly occurs in malignancies and contributes to many aspects of tumorigenesis. A number of molecular chaperones are up-regulated in cancer in response to ER stress, including heat shock proteins (such as GPR78 and GPR94) and lectin-like chaperones (such as calnexin and calreticulin) [34, 35]. It is also possible that the increased Tn antigen regulates T-synthase/Cosmc production via a feedback loop.

Our findings in CRC cell lines and specimens suggest more complicated mechanisms for Tn expression. Protein glycosylation is spatiotemporally controlled by glycosyltransferases and other enzymes. It is possible that in Tn(+) CRC specimens, the initiating enzymes ppGalNAc-Ts may be abnormally expressed or mislocalized. Several ppGalNAc-Ts including T1, T3, T6, and T13 are reported to be elevated in human cancers [36–39]. Since these ppGalNAc-Ts have overlapping yet distinct substrate specificities, their up-regulation may increase GalNAc linkage to Ser/Thr residues or generate aberrantly glycosylated proteins at novel or cryptic sites, resulting in unusual conformations that may not be recognized or modified by T-synthase or core 3 synthase (B3GnT6) [40]. For example, it was reported that translocation of ppGalNAc-Ts from Golgi to endoplasmic reticulum (ER) was associated with high Tn levels in breast cancer, although the studies did not mechanistically explain the production of cell surface Tn antigen [41]. It is also possible that the in vivo ‘functional’ activity of T-synthase may be comprised in CRC samples due to mislocalization or enzyme inhibition in situ. Cancer cells often have lost the intact structure of the Golgi apparatus and exhibit an altered pH [42]. T-synthase, even correctly folded, may be mislocalized to aberrant compartments of the Golgi where access to newly synthesized Tn antigen-containing glycoproteins is compromised. Our current enzymatic assay and antibodies cannot assess activity of the T-synthase protein in situ. Furthermore, the access of T-synthase to the Tn antigen could be blocked either by an endogenous inhibitor, as observed for GnT1IP-L towards MGAT1 [43], or by a Tn antigen-binding molecule presented in cancer cells. In addition, Tn antigen expression may be caused by dysregulation of other enzymes, such as core 3 synthase (B3GnT6). B3GnT6 converts the Tn antigen paralleled to the T-synthase/Cosmc complex in the GI tract [12]. It suppresses the metastatic potential and was reported to be down-regulated in colon carcinoma [44]. Mice lacking core 3-derived O-glycans had increased susceptibility to colitis and colorectal tumors [45]. Although we did not observe any mutation of *B3GNT6* coding region and its transcript level was undetectable in most CRC samples examined, it is still unknown whether B3GnT6 loses its function in Tn-positive CRCs. It is possible that dysregulation of core 3 synthase could compromise activity of T-synthase, leading to enhanced Tn expression. Finally, the in vivo microenvironment may affect Tn antigen expression. In patients, cancer cells grow in a microenvironment influenced by the oxygen level, cytokines, cellular polarity, and stromal contact. Recent studies showed that cytokine-initiating signaling may regulate the Tn antigen, and hypoxia promotes the STn antigen in bladder cancer [46, 47]. In addition, hypoxia also up-regulates the transcription of UGT-1 in CRC cells, which transports UDP-Gal and probably UDP-GalNAc, suggesting that hypoxia may affect nucleotide sugar pools to modulate glycosylation [48, 49]. The difference between in vivo and in vitro environment may explain why most established cancer cell lines do not express the Tn neoantigen. Thus, it may be that a loss of functional T-synthase, through one or more putative pathways discussed above, leads to Tn expression, which further serves as a substrate of ST6GALNAC1 that was elevated in many CRC samples. Some of these hypotheses are currently under investigation.

## Conclusions

In summary, our results suggest that loss of T-synthase/Cosmc due to genetic and epigenetic inactivation of *Cosmc* may be responsible for Tn expression in human cancer cell lines and pancreatic cancer, while alternative mechanisms exist in Tn-positive colorectal cancers.

## Additional Files

Additional file 1: Genetic alterations of *Cosmc* in cancer cell lines and specimens. A Table containing the names of the cell line used and a summary of expression and genetic details including references. (DOCX 26 kb)

Additional file 2: Single nucleotide polymorphisms (SNPs) used in the loss of heterozygosity (LOH) analysis. A Table containing specific SNPs and primer and PCR details. (DOCX 16 kb)

Additional file 3: Characterization of the Tn neoantigen and T-synthase in human colorectal cancer (CRC) cell lines. A Figure containing 3 panels of data: a, expression levels of the Tn antigen and T-synthase as shown by Western Blot. b, A chart of T-synthase enzyme activities in CRC cell lines. c, Representative images of immunofluorescence of the Tn antigen in CRC cell lines. (DOCX 268 kb)

Additional file 4: Expression of the STn antigen in LS 180 and HCT8 subpopulations. A Figure containing 2 panels of immunofluorescence data on STn expression in cell lines. (DOCX 307 kb)

Additional file 5: Summary of the expression of Tn and STn antigens and T-synthase/Cosmc in human colorectal cancer samples. A Table containing the antigen expression and changes in expression in all of the anonymous case studies. (DOCX 21 kb)

Additional file 6: Expression of the blood group A (BGA) antigen in human CRCs. A Figure containing the blood group A antigen expression in two case studies. (DOCX 307 kb)

## Abbreviations

C1GALT1: UDP-Gal:GalNAcα1-O-Ser/Thr glycopeptide β3-galactosyltransferase
BGA: Blood group A antigen
C1GalT1C1: C1GALT1-Specific Chaperone 1
CRC: Colorectal cancer
FACS: Fluorescence-activated cell sorting
IHC: Immunohistochemistry
IS: IHC score
LOH: Loss of heterozygosity
MSI: Microsatellite instability
RFU: Relative fluorescence units
STn: Sialyl Tn
TM: Transitional mucosa
