# Supplementary material for: Differential expression of Cosmc, T-synthase and mucins in Tn-positive colorectal cancers
Source: BMC Cancer. 2018 Aug 16;18:827. doi: 10.1186/s12885-018-4708-8 (PMC6097208; doi:10.1186/s12885-018-4708-8)
Supplement: Supplementary file 1 — Genetic alterations of Cosmc in cancer cell lines and specimens. A Table containing the names of the cell line used and a summary of expression and genetic details including references. (DOCX 26 kb) [file 12885_2018_4708_MOESM1_ESM.docx]

**Additional file 1** Genetic alterations of *Cosmc* in cancer cell lines and specimens

| **Cancer/cell lines** | **Tn/STn expression** | **Genetic alterations in *COSMC*** | **Change in COSMC protein** | **Refs** |
| --- | --- | --- | --- | --- |
| **Human** |  |  |  |  |
| Jurkat (T cell leukemia) | Tn | delT473 | ORF shift: truncated protein | [1] |
| LOX (melanoma) | Tn | Promoter deletion | No protein made | [2] |
| Tn4 (Immortalized B cell) | Tn | Promoter silencing | No protein made | [3] |
| LSC (colorectal cancer) | Tn & STn | insT53 | ORF shift: 28AA peptide | [2] |
| LS174T Tn(+)-I (colorectal cancer) | Tn & STn | delA482 | ORF shift: truncated protein | [2] |
| LS174T Tn(+)-II (colorectal cancer) | Tn & STn | G553T | C185X: truncated protein | [2] |
| HT-29 (colorectal cancer) | Tn & STn | Deletion | No protein made | [4] |
| LS 180 Tn(+) (colorectal cancer) | Tn & STn | delT473 | ORF shift: truncated protein | this study |
| HCT8 Tn(+) (colorectal cancer) | Tn | delA482 | ORF shift: truncated protein | this study |
| Human cervical cancer | Tn & STn | Deletion (LOH) | No protein made | [2] |
| Human pancreatic cancer | Tn & STn | Promoter silencing | Reduced protein level | [5] |
| **Mouse** |  |  |  |  |
| Neuro2a (neuroblastoma) | Tn | G301T | E101X: truncated protein | [6] |
| Fibrosarcoma | Tn & STn | delC509-A587 | T170-L195 deletion | [6] |

X indicates stop codon.

[1] Ju T, Cummings RD. A unique molecular chaperone Cosmc required for activity of the mammalian core 1 beta 3-galactosyltransferase. Proc Natl Acad Sci U S A 2002;99:16613-8.

[2] Ju T, Lanneau GS, Gautam T, Wang Y, Xia B, Stowell SR, et al. Human tumor antigens Tn and sialyl Tn arise from mutations in Cosmc. Cancer Res 2008;68:1636-46.

[3] Mi R, Song L, Wang Y, Ding X, Zeng J, Lehoux S, et al. Epigenetic silencing of Cosmc in human leukocytes expressing Tn antigen. J Biol Chem 2012.

[4] Yu X, Du Z, Sun X, Shi C, Zhang H, Hu T. Aberrant Cosmc genes result in Tn antigen expression in human colorectal carcinoma cell line HT-29. International journal of clinical and experimental pathology 2015;8:2590-602.

[5] Radhakrishnan P, Dabelsteen S, Madsen FB, Francavilla C, Kopp KL, Steentoft C, et al. Immature truncated O-glycophenotype of cancer directly induces oncogenic features. Proc Natl Acad Sci U S A 2014;111:E4066-75.

[6] Schietinger A, Philip M, Yoshida BA, Azadi P, Liu H, Meredith SC, et al. A mutant chaperone converts a wild-type protein into a tumor-specific antigen. Science 2006;314:304-8.
