# Supplementary material for: Differential expression of Cosmc, T-synthase and mucins in Tn-positive colorectal cancers
Source: BMC Cancer. 2018 Aug 16;18:827. doi: 10.1186/s12885-018-4708-8 (PMC6097208; doi:10.1186/s12885-018-4708-8)
Supplement: Supplementary file 2 — Single nucleotide polymorphisms (SNPs) used in the loss of heterozygosity (LOH) analysis. A Table containing specific SNPs and primer and PCR details. (DOCX 16 kb) [file 12885_2018_4708_MOESM2_ESM.docx]

**Additional file 2** Single nucleotide polymorphisms (SNPs) used in the loss of heterozygosity (LOH) analysis

| **SNP ID** | **Primers** | **Primer sequence** | **Size of PCR product (bp)** | **Location in the gene** |
| --- | --- | --- | --- | --- |
| ***Cosmc*** |  |  |  |  |
| *rs5910941* | forward | 5’-TTTTCTTTTCCAGCGGGC-3’ | 463 | upstream |
|  | reverse | 5’-GGGAGAGTGAGCAAGACTCC-3’ |  |  |
| *rs3810744* | forward | 5'-CAGTGGCTTGCGTTTTGG-3' | 529 | Promoter |
|  | reverse | 5'-GCACGGGTTTCCTCTCAC-3' |  |  |
| *rs5957424* | forward | 5’-TTTTTGCCTCTGTGATGGG-3’ | 124 | intron 1 |
|  | reverse | 5’-TGATGGTGATCCTACTCCTGG-3’ |  |  |
| *rs?* | forward | 5’-CTCTGCCTCCTGGTTTCAAG-3’ | 477 | downstream |
| *rs17327439* | reverse | 5’-TCTAACACTCTATGCGGACTCAC-3’ |  |  |
| *rs5910940* |  |  |  |  |
| ***T-synthase*** |  |  |  |  |
| *rs4720725* | forward | 5’-CACGTGCAGAGGTACTGGAA-3’ | 359 | intron 1 |
| *rs4720726* | reverse | 5’-GTGCCCTGTGCTATCCTCAG-3’ |  |  |
| *rs11336626* |  |  |  |  |
| *rs4724960* | forward | 5’-TGAAAGCTGTCAGACCTGGA-3’ | 274 | intron 1 |
|  | reverse | 5’-GGAAGCGTGAAACCTGAAAG-3’ |  |  |
| *rs11767985* | forward | 5’-CCTTTGGTATCTGCAGGGTC-3’ | 190 | intron 1 |
| *rs11767988* | reverse | 5’-AAACCCCCATGACACACAAT-3’ |  |  |
| *rs7811763* | forward | 5’-AAAATTGGTTGTGGCAGAGG-3’ | 470 | intron 1 |
| *rs2108784* | reverse | 5’-AACCATTCATGAGGGCAGAG-3’ |  |  |
| *rs2108783* |  |  |  |  |
| *rs2159191* |  |  |  |  |
| *rs7794210* | forward | 5’-GGAGCCTGAAGTTTTGTTGG-3’ | 413 | intron 3 |
| *rs7790522* | reverse | 5’-CCCAAATTTTGTGGGCTAGT-3’ |  |  |
| *rs11764290* | forward | 5’-AGGTTGGTGCTATTTTGGGA-3’ | 191 | downstream |
|  | reverse | 5’-TGCTATGACACTCAGTACTGCTAAA-3’ |  |  |
