# Supplementary material for: Differential expression of Cosmc, T-synthase and mucins in Tn-positive colorectal cancers
Source: BMC Cancer. 2018 Aug 16;18:827. doi: 10.1186/s12885-018-4708-8 (PMC6097208; doi:10.1186/s12885-018-4708-8)

**Additional file 3** Characterization of the Tn neoantigen and T-synthase in human colorectal cancer (CRC) cell lines. **a**, expression levels of the Tn antigen and T-synthase determined by WB. SW1116 cells were examined at both low (L) and high (H) cell densities. LS174T-Tn(+)-II cells expressed Tn and had no T-synthase. All other CRC cell lines expressed detectable T-synthase but no Tn. Names of cell lines are listed at the top. Protein standards are labeled at the left, and antibodies at the right. **b**, T-synthase specific enzyme activities in CRC cell lines. Enzyme activities were determined in triplicate, and error bars represent the standard error of the mean (SEM). Names of cell lines are listed at the bottom. **c**, representative images of immunofluorescence of the Tn antigen in CRC cell lines LS 180, HCT8, SW480, and SW1116. In each line, a small percentage of cells express detectable Tn antigen (green) on the cell surface. Nuclei were counterstained with DAPI (blue). All scale bars are 50 μm


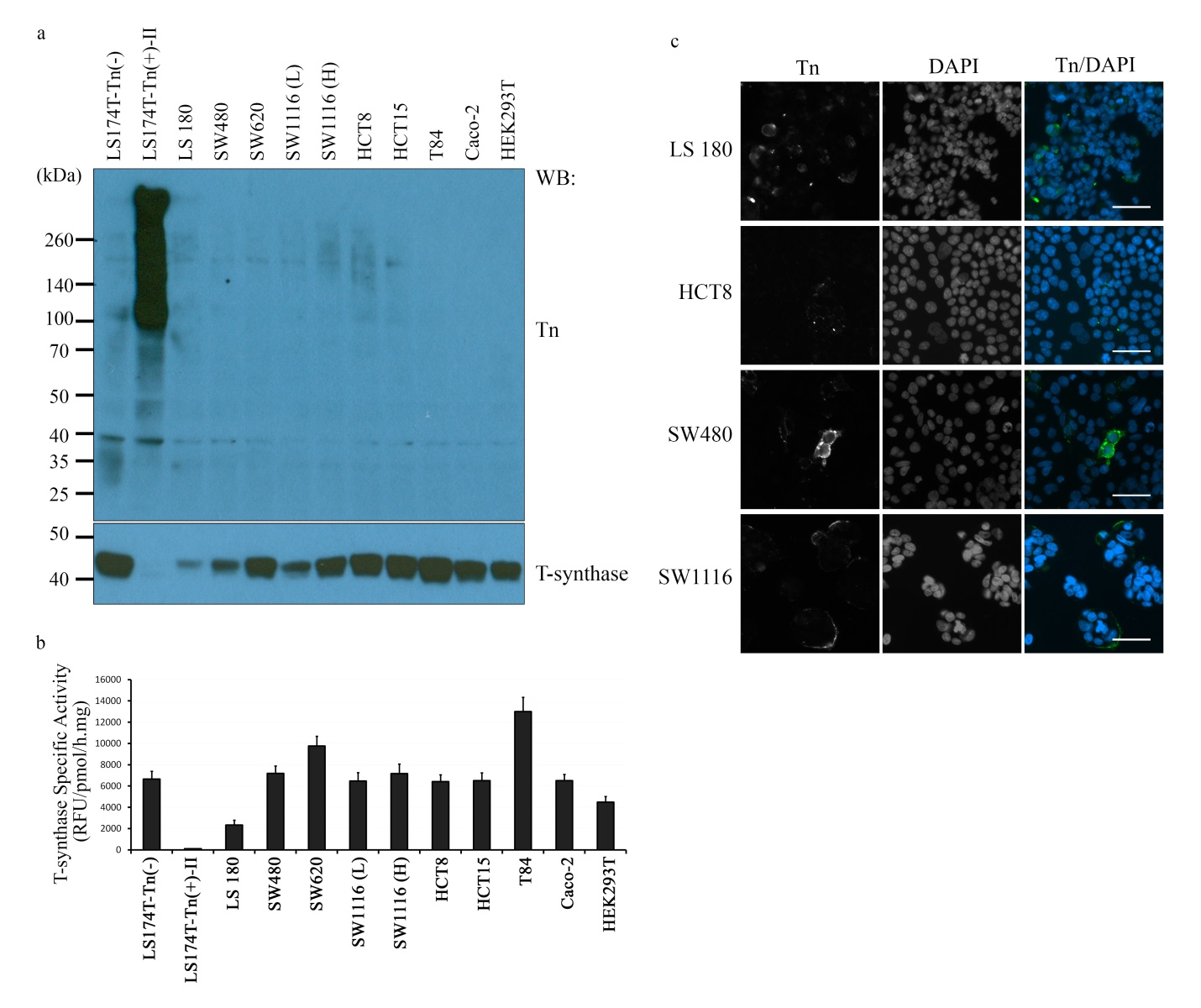

Supplement: Supplementary file 3 — Characterization of the Tn neoantigen and T-synthase in human colorectal cancer (CRC) cell lines. A Figure containing 3 panels of data: a, expression levels of the Tn antigen and T-synthase as shown by Western Blot. b, A chart of T-synthase enzyme activities in CRC cell lines. c, Representative images of immunofluorescence of the Tn antigen in CRC cell lines. (DOCX 268 kb) [file 12885_2018_4708_MOESM3_ESM.docx]
