# Supplementary material for: Differential expression of Cosmc, T-synthase and mucins in Tn-positive colorectal cancers
Source: BMC Cancer. 2018 Aug 16;18:827. doi: 10.1186/s12885-018-4708-8 (PMC6097208; doi:10.1186/s12885-018-4708-8)
Supplement: Supplementary file 4 — Expression of the STn antigen in LS 180 and HCT8 subpopulations. A Figure containing 2 panels of immunofluorescence data on STn expression in cell lines. (DOCX 307 kb) [file 12885_2018_4708_MOESM4_ESM.docx]

**Additional file 4** Expression of the STn antigen in LS 180 and HCT8 subpopulations. **a**, immunofluorescence of the STn antigen (green) in LS 180 parental, Tn(-) and Tn(+) cells. LS 180 parental cells contained a few STn-positive cells. Compared to LS 180 Tn(-) cells, Tn(+) cells expressed robust STn antigen on the cell surface. **b**, immunofluorescence of the STn antigen (green) in HCT8 parental, Tn(-) and Tn(+) cells. All HCT8 cells are negative for STn staining. Nuclei were counterstained with DAPI. Cell populations are listed at left. All scale bars are 50 μm

**
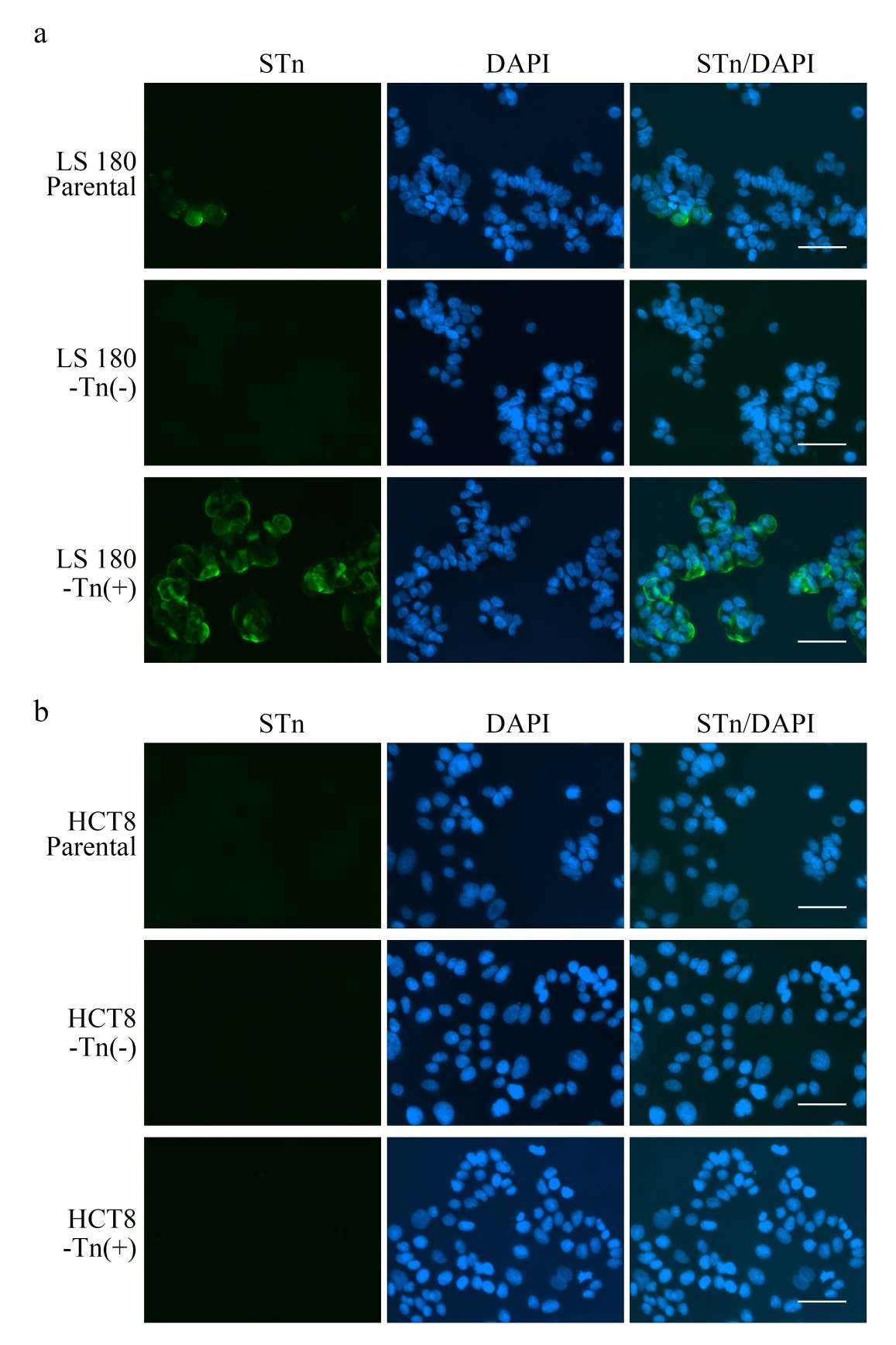
**
