# Supplementary material for: Differential expression of Cosmc, T-synthase and mucins in Tn-positive colorectal cancers
Source: BMC Cancer. 2018 Aug 16;18:827. doi: 10.1186/s12885-018-4708-8 (PMC6097208; doi:10.1186/s12885-018-4708-8)
Supplement: Supplementary file 5 — Summary of the expression of Tn and STn antigens and T-synthase/Cosmc in human colorectal cancer samples. A Table containing the antigen expression and changes in expression in all of the anonymous case studies. (DOCX 21 kb) [file 12885_2018_4708_MOESM5_ESM.docx]

**Additional file 5** Summary of the expression of Tn and STn antigens and T-synthase/Cosmc in human colorectal cancer samples^a^

| **Case #** | **Blood**  **type** | **IS of Tn** | | **IS of STn** | | **Cosmc** | | | **T-synthase** | | |
| --- | --- | --- | --- | --- | --- | --- | --- | --- | --- | --- | --- |
|  |  | **N** | **T** | **N** | **T** | **locus** | **mRNA** | **protein** | **mRNA** | **protein** | **activity** |
| 1 | B/O | 0 | 250 | 0 | 130 |  | - | Up | Up | Up | Up |
| 2 | A/AB | 100* | 170 | 40* | 190 | - | Up | Up | Up | Up | Up |
| 3 | B/O | 0 | 130 | 0 | 240 | - | - | Down | Down | Down | - |
| 4 | U | 0 | 160 | 0 | 160 | - | - | Up | Up | Up | Up |
| 5 | U | 20* | 220 | 10* | 210 |  | Up | Up | Up | Up | Up |
| 6 | B/O | 10* | 90 | 0 | 70 |  | Up | Up | Up | Up | Up |
| 7 | B/O | 0 | 90 | 0 | 60 |  | Up | Up | Up | - | Up |
| 8 | B/O | 0 | 180 | 0 | 110 |  |  | Down |  | Up | Up |
| 9 | A/AB | 150 | 200 | 0 | 140 | - | Up | Up | Up | Up | Up |
| 10 | A/AB | 20* | 150 | 20* | 150 | LOH | - | Up | Up | - | - |
| 11 | A/AB | 5* | 240 | 40* | 270 | LOH | Up | Up | Up | - | Up |
| 12 | B/O | 5* | 10 | 0 | 0 | LOH |  | Up |  | - | Up |
| 13 | A/AB | 280 | 240 | 0 | 180 | LOH |  | Up |  | - | Up |
| 14 | A/AB | 170 | 220 | 0 | 180 | - | Down | - | Up | Up | - |
| 15 | A/AB | 70 | 170 | 60 | 140 | - | Up | Up | Up | Up | Up |
| 16 | A/AB | 90 | 130 | 0 | 50 |  | - | Up | - | Up | - |
| 17 | B/O | 5* | 120 | 0 | 120 |  |  | Up |  | Up | - |
| 18 | A/AB | 0 | 200 | 0 | 170 |  |  | Up |  | Up | - |
| 19 | B/O | 10* | 110 | 0 | 40 | LOH |  | Up |  | Up | Up |
| 20 | A/AB | 260 | 130 | 0 | 90 | - |  | Up |  | Up | - |
| 21 | B/O | 0 | 80 | 0 | 30 |  |  | - |  | - | - |
| 22 | B/O | 5* | 50 | 0 | 0 |  |  | Up |  | Up | Up |
| 23 | A/AB | 30* | 220 | 5* | 160 |  | Down | Down | Down | Down | Down |
| 24 | B/O | 30* | 240 | 0 | 20 |  |  | - |  | - | - |
| 25 | A/AB | 20 | 270 | 0 | 150 | LOH | Up |  | Up |  |  |
| 26 | B/O | 10* | 150 | 0 | 110 | LOH |  |  |  |  |  |
| 27 | A/AB | 5* | 190 | 5* | 140 | LOH |  |  |  |  |  |
| 28 | A/AB | 40 | 245 | 0 | 40 |  |  |  |  |  |  |
| 29 | A/AB | 10* | 20* | 0 | 0 |  |  |  |  |  |  |
| 30 | B/O | 0 | 90 | 0 | 10 |  |  |  |  |  |  |
| 31 | A/AB | 0 | 50 | 0 | 10 |  |  |  |  |  |  |
| 32 | B/O | 20* | 160 | 0 | 200 |  |  |  |  |  |  |
| 33 | B/O | 5* | 150 | 0 | 20 |  |  |  |  |  |  |
| 34 | A/AB | 300 | 140 | 0 | 10 |  |  |  |  |  |  |
| 35 | A/AB | 10* | 170 | 0 | 40 |  |  |  |  |  |  |
| 36 | B/O | 10* | 100 | 0 | 50 |  |  |  |  |  |  |
| 37 | B/O | 15* | 170 | 0 | 100 |  |  |  |  |  |  |
| 38 | B/O | 0 | 130 | 0 | 120 |  |  |  |  |  |  |
| 39 | A/AB | 5* | 150 | 10* | 160 |  |  |  |  |  |  |
| **All** | **18 B/O**  **19**  **A/AB**  **2 U** | **7/39 Tn+** | **37/39**  **Tn+** | **1/39 STn+** | **27/39 STn+** | **8/15** | **8/15 Up**  **2/15 Down** | **18/24 Up**  **3/15 Down** | **12/15 Up**  **2/15 Down** | **15/24 Up**  **2/24 Down** | **14/24 Up**  **1/24 Down** |

^a^: Blood group antigens were determined by immunostaining with an antibody against blood group A antigen. Asterisks (*) indicate intracellular staining. For Tn and STn staining on cell surface, a sample is considered to be positive when the IS is 50 or greater. For mRNA and protein levels and T-synthase activities, the threshold was set up as 2-fold change, and a minus indicates either no change or that the changes is less than 2 folds. Unexamined samples are left in blank. IS, IHC score. N, matched adjacent normal tissue. T, tumor tissue. LOH, loss of heterozygosity. U, unknown. Gender totals: 14 males, 16 females, 9 U; gender identity is not given specifically for each case in order to maintain anonymity.
