# Supplementary material for: Differential expression of Cosmc, T-synthase and mucins in Tn-positive colorectal cancers
Source: BMC Cancer. 2018 Aug 16;18:827. doi: 10.1186/s12885-018-4708-8 (PMC6097208; doi:10.1186/s12885-018-4708-8)

**Additional file 6** Expression of the blood group A (BGA) antigen in human CRCs. Positive staining of BGA antigen was shown in 2 cases of matched normal and tumor specimens by IHC. The areas are identical to those shown in Fig.4a. In all areas, BGA was expressed in the stroma. All scale bars are 100 μm


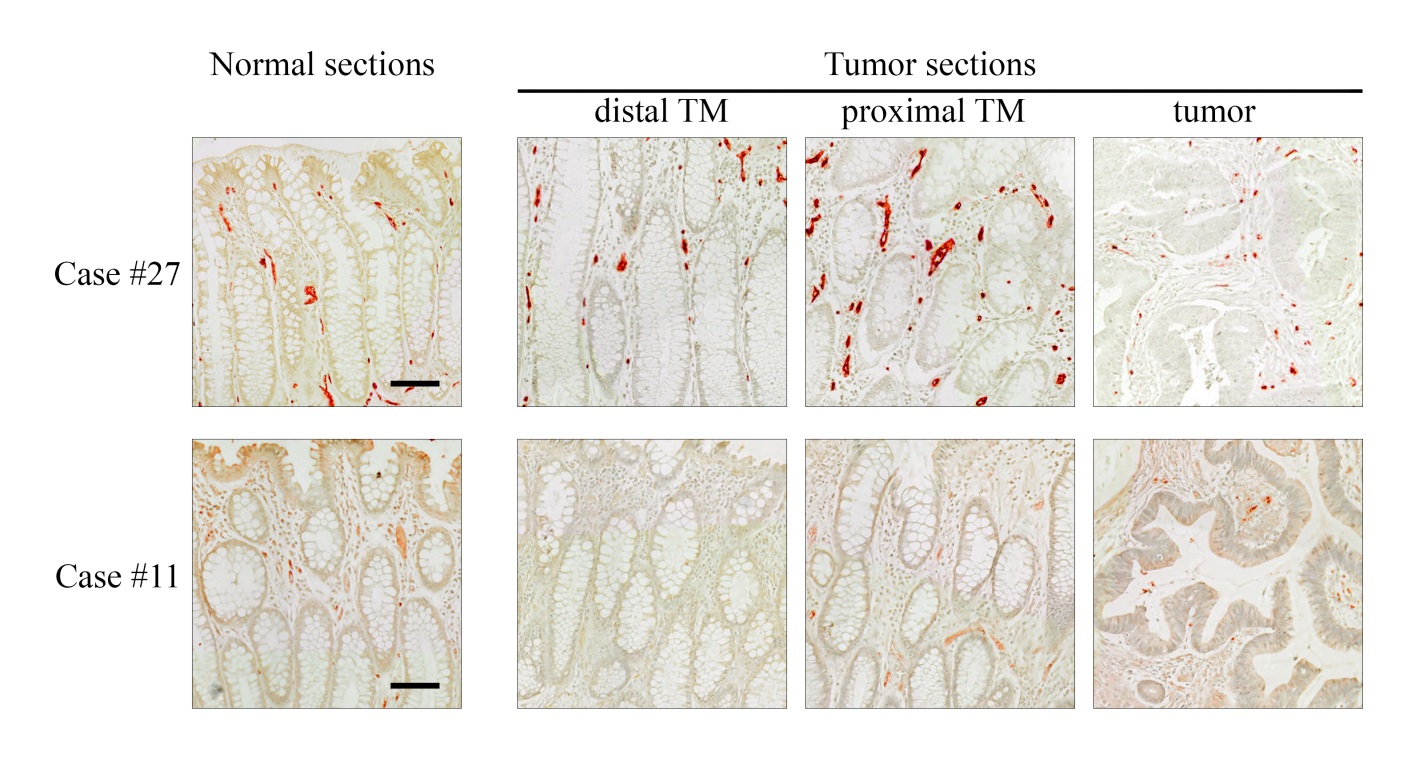

Supplement: Supplementary file 6 — Expression of the blood group A (BGA) antigen in human CRCs. A Figure containing the blood group A antigen expression in two case studies. (DOCX 307 kb) [file 12885_2018_4708_MOESM6_ESM.docx]
